# Critical care capacity in Africa: postpandemic ICU capacity, service readiness and patient profiles across public and private hospitals in Ethiopia

**DOI:** 10.1136/bmjgh-2025-021281

**Published:** 2026-03-24

**Authors:** Fitsum K Belachew, Tesfay Yohannes Ambese, Degisew Dersso, Selam Daniel, Azeb Demelash, Kalkidan Kifle, Wagari Tuli Nora, Elubabor Buno, Emnet Tesfaye Shimber, Yared Boru, Rupert Pearse, Menbeu Sultan, on behalf of ENCCA collaborators, Desta Kassaye, Kerebih Hailemariam, Workineh Aniley, Ali Seid, Chanyalew Abdeta, Moti Dejene, Yonas Belachew, Solomon Addise, Dereje Hailu, Yonatan Tesema, Kejela Mosisa, Eyuel Teshome, Ambawu Siyum, Gashaw Wkidan, Hmariam Zekarias, Melke Mare, Hirut Tolasa, Feregzh Dinku, Abeba Tegegn, Ali Mohammed, kedir Hussen, Ibrahim Aliye, Ahmed Adem, Andargie Atnaf, Sisay Nigusu, Kibret Engocha, Shumet Gathaye, Astazbew Tehuala, Siquar Zinabu, Getie Demelash, Getasew Misganaw, Dinku Yegnasew, Bruk Tsegaye, Wondissew Admasu, Habtamu Nahusenay, Begosew Yeshiwas, Awoke Warkaw, Asnakew Alem, Tesfaye Ayenew, Tirusew Getie, Kemal Adem, Biruktie GebreAregawi, Biftu Gudisa, Tariku Lenchamo, Tadele Temesgen, Yared Hailu, Zelalem Nigussie, Eyuel Tewodros, Teka Kemal, Ashanif Temsgne, Solomon Abera, Yonas Alamrew, Biruk Tizazu, Seid Ali, Beyazilign Alemu, Kananisa Layo, Bayisa Guteta, Amde Getahun, Girma Mesfin, Tamirat Tesfaye, Garoma Gamachu, Feleke Ledago, Gamachis Amenu, Dawit Legesse, Canyalew Abdeta, Abdikadir A Abdi, Nimcan A Abdi, Salamoon warqu, Hassen A Hussein, Asaminew Tasew, kindalem Worku, Aytnew Debebe, Tariku Tadese, Getachew Samuel, Woineshet Akinaw, Wondimu Dori, Ashenafi Anjulo, Brihanu Anjulo, Edgete Lemma, Alemayew Yesehaq, Doctor Antenhe Berhane, Tewodros Gobezay, Tsegay G Mikael, Merhawit Abreha, Yilikal Gebru

**Affiliations:** 1 Network for Perioperative and Critical Care (N4PCc), Asrat Woldyes Health Sciences Campus, Debere Berhan University, Debre Berhan, Ethiopia; 2 Department of Statistical and Data Governance, Global Partners for Improving Surgical System (GPISS), Project Network for Perioperative and Critical Care (GPISS-N4PCc), Addis Ababa, Ethiopia; 3 Medical Services, Ethiopia Ministry of Health, Addis Ababa, Ethiopia; 4 Department of Anesthesia, St. Peter Specialized Hospital, Addis Ababa, Ethiopia; 5 Department of Emergency and Critical Care, Addis Ababa University College of Health Sciences, Addis Ababa, Ethiopia; 6 Department of Emergency Medicine and Critical Care, Hawassa University, Awassa, SNNPR, Ethiopia; 7 Network for Perioperative and Critical Care, Asrat Woldeyes Health Sciences Campus, Debre Berhan University, Debre Birhan, Amhara, Ethiopia; 8 Intensive Care Medicine, Queen Mary University of London, London, UK; 9 Emergency and Critical Care, St Paul’s Hospital Millennium Medical College, Addis Ababa, Oromia, Ethiopia; 10 ENCCA collaborators, Addis Ababa, Ethiopia

**Keywords:** Africa, Global Health, Health systems

## Abstract

**Background:**

The COVID-19 pandemic exposed critical care disparities in low-resource settings. In Ethiopia, intensive care unit (ICU) expansion had begun prior to COVID-19 but accelerated during the pandemic with substantial new investment. This study provides the first nationwide assessment of ICU capacity, service readiness and patient characteristics across both public and private hospitals.

**Methods:**

A cross-sectional survey was conducted in 2024 across all Ethiopian hospitals with operational ICUs, using site visits and standardised tools to assess facility capacity, staffing, equipment and preparedness. Patient-level data were collected for admissions on the survey day. Results were compared with pre-COVID-19 data to evaluate progress.

**Findings:**

A total of 159 hospitals (117 public, 42 private) were surveyed, encompassing 1028 ICU beds. Public ICU facilities increased from 51 to 117 since COVID-19, with beds rising from 324 to 762. Private facilities added 266 beds (25.9% of the total). Improvements included 24/7 ICU-trained physician availability (52.1% vs 29.0% pre-COVID-19) and disaster preparedness plans (21.4% vs 6.0%). Persistent gaps were evident in advanced haemodynamic monitoring (available in <10 facilities) and organ support (9/117 public ICUs). Among 279 admissions (mean age 39.1 years; 55.2% male), neurological (32.1%) and respiratory (25.8%) conditions predominated, with sepsis observed in 29.4% of patients. Hypertension (25.1%) and diabetes (17.2%) were common comorbidities.

**Interpretation:**

Ethiopia’s ICU capacity has tripled since 2019, with a more balanced regional distribution and improved workforce coverage; however, deficits in advanced monitoring, organ support and referral coordination limit the system’s readiness. The high burden of sepsis among ICU patients highlights systemic gaps in early recognition and supportive care; this supports scaling Essential Emergency and Critical Care as a foundational platform, alongside targeted improvements in infection management and antimicrobial stewardship. Strengthening public-private integration, standardised referral systems and cost-effective, high-impact interventions will be key to improving equity and outcomes in Ethiopia and comparable settings across sub-Saharan Africa and other low-resource contexts.

**WHAT IS ALREADY KNOWN ON THIS TOPIC**

- Before the COVID-19 pandemic, Ethiopia had only 0.3 intensive care unit (ICU) beds per 100 000 population, among the lowest globally and most available evidence focused solely on public-sector ICUs.
- Private facilities, despite their essential role in urban settings, were largely absent from national assessments, and a limited workforce and limited equipment constrained critical care delivery.
- Although the pandemic prompted major investments in ICU infrastructure and training across sub-Saharan Africa, the scale, distribution and sustained impact of these efforts remain poorly quantified.

**WHAT THIS STUDY ADDS**

- This study provides the first nationwide, post-COVID-19 assessment of ICU capacity in Ethiopia across both public and private hospitals, offering insights relevant to similar low-resource African settings.
- It shows that ICU capacity has expanded substantially since the pandemic, with hospitals providing ICU services and total bed numbers nearly tripling, while persistent gaps remain in advanced monitoring, organ support and emergency preparedness.
- The study also presents the first national patient-level snapshot of ICU case mix, revealing a predominantly young population with high burdens of neurological, respiratory and sepsis-related conditions.

**HOW MIGHT THIS STUDY AFFECT RESEARCH, PRACTICE OR POLICY**

- These findings provide a national baseline to inform strategic critical care planning in Ethiopia and other African countries pursuing long-term strengthening of their critical care systems.
- The results support prioritising essential emergency and critical care, workforce development and functional referral systems alongside selective ICU expansion.
- Including private facilities highlights opportunities for structured public-private integration and more comprehensive national ICU surveillance.

## Background

Critical care, which includes timely interventions for patients with life-threatening yet potentially reversible conditions, is a vital part of health systems.^1 2^ Intensive care units (ICUs) play a central role by delivering continuous monitoring and advanced organ support for critically ill patients.^3^ However, ICU capacity varies widely across countries. While some low- and middle-income countries (LMICs), such as South Sudan and Nauru, have no ICU beds, others like Kazakhstan report 21.3 beds per 100 000 population.^4 5^ In Africa, ICU bed availability remains low, with only Egypt, South Africa and Seychelles exceeding five beds per 100 000 population.^4 5^

LMICs face a disproportionate burden of critical illness and markedly higher mortality rates compared with high-income countries.^6 7^ These disparities, highlighted further by the COVID-19 pandemic, are driven by limited ICU infrastructure, shortages of trained personnel and fragmented referral systems.^8–10^ Resource constraints contribute to insufficient critical care delivery and to delayed recognition and treatment of patient deterioration, factors that compound poor outcomes, especially in sub-Saharan Africa.^11–13^

In Ethiopia, a 2020 national review revealed substantial gaps in ICU services, including limited beds, a lack of equipment and workforce shortages.^14^ Moreover, the assessment focused only on public-sector ICUs and did not capture private facilities or patient-level data.^14^ In response, the Ethiopian Ministry of Health, along with its partners, launched several initiatives to expand ICU capacity, improve oxygen access and strengthen workforce training.^15 16^ The national impact of these interventions, however, remains unquantified. Complementary evidence from the national Ethiopian Surgical Outcomes Study (Ethio-SOS) highlighted perioperative outcomes, with nearly one in five surgical patients developing complications and 3.5% requiring ICU admission.^17^ While Ethio-SOS highlighted perioperative outcomes, the present study completes the national picture by assessing ICU capacity, readiness and patient profiles across public and private facilities.

Building on these earlier initiatives, it is important to note that ICU expansion in Ethiopia did not begin with COVID-19. National strategies such as the Health Sector Development Plan V (HSDP) and the Saving Lives through Safe Surgery (SaLTS) initiative had already prioritised critical care strengthening.^18 19^ The pandemic, however, accelerated these efforts through unprecedented investment, with approximately US$154 million mobilised nationally, including support for ICU infrastructure, oxygen generation and workforce training. The current assessment, therefore, reflects both the continuation of long-term reforms and the step-change in investment during COVID-19.

This study addresses that gap by conducting a nationwide cross-sectional assessment of ICU capacity and patient case mix in public and private hospitals across Ethiopia. By capturing both facility-level and patient-level data, this analysis provides a comprehensive view of Ethiopia’s critical care landscape and offers insights relevant to scaling up essential emergency and critical care services in other low-resource settings.

## Method

### Study design

**Figure 1:**
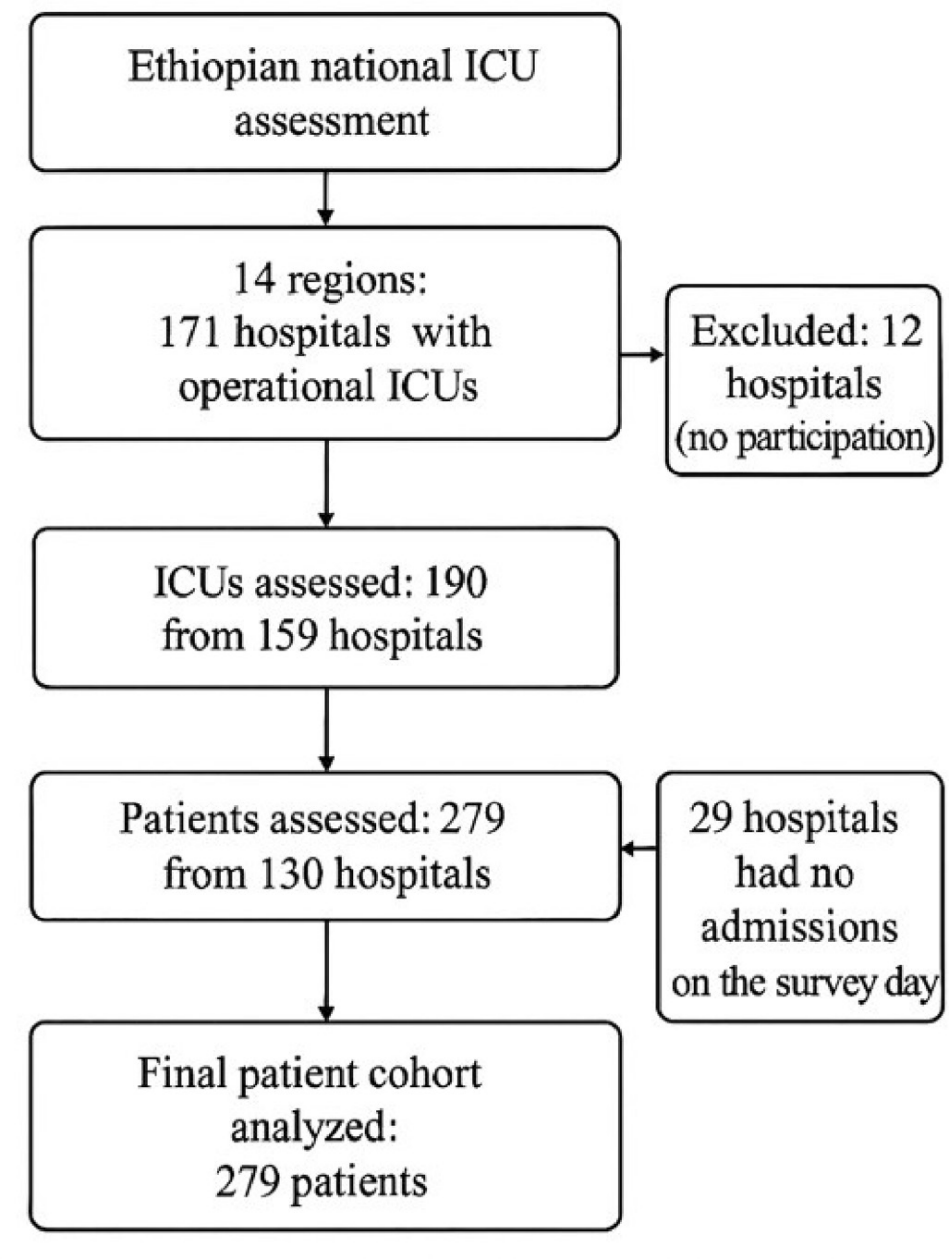
Flow diagram of hospital, intensive care unit (ICU) and patient inclusion in the Ethiopian National Critical Care Assessment (ENCCA). Of 171 hospitals with operational ICUs, 159 (93%) were surveyed. Twelve hospitals did not participate, and 29 had no patients admitted on the survey day. A total of 190 ICUs and 279 patients were included in the final analysis.

This study used a nationwide, cross-sectional study design to assess ICU services across Ethiopia. Data collection took place from March to October 2024, and tried to involve all public and private healthcare facilities with operational ICUs. The study was designed, analysed and reported in accordance with the Strengthening the Reporting of Observational Studies in Epidemiology guidelines for cross-sectional studies.^20^ A flow diagram (figure 1) shows hospital, ICU and patient inclusion and exclusion.

### Study tool design

The survey tool was adapted from the World Federation of Intensive Care Medicine (WFICC) guidelines and a 2019 survey. It was further refined to address limitations identified in earlier data collection rounds.^3 14^ This was a nationwide cross-sectional study with a census approach, in which all hospitals with operational ICUs were approached. Therefore, no formal sample size calculation was required. These units had previously been made aware of WFICC criteria through earlier national assessments, and facilities that did not fulfil this minimum threshold generally did not self-identify as ICUs. The survey tool comprised four forms designed to comprehensively evaluate ICU practices and patient care. These forms assessed key areas critical to understanding ICU operations and their capacity to deliver care:

- ICU infrastructure: design, space utilisation and layout.
- Staffing: availability and qualifications of physicians, nurses and allied health professionals, including nurse-to-patient ratios.
- Resources and equipment: monitoring capabilities and support for organ function.
- Educational and training opportunities: presence of formal professional development programmes and the ICU’s role as a training centre.
- Outreach and integration: ICU services linked to emergency departments, hospital wards and follow-up care for discharged patients.
- Research and quality improvement capacity: ICU care providers’ involvement in advancing care standards and outcomes via research and quality improvement.
- Scalability: ability to respond and expand during disasters.

### Each of the survey forms was tailored to address these areas systematically

- General information form: collected hospital-level data, including hospital type, total number of available beds and the number of ICUs.
- ICU-specific form: captured detailed information about ICU operations, including staffing levels, equipment availability and the presence of standardised protocols. For hospitals with multiple ICUs, this form was completed for each unit individually.
- ICU observation form: focused on direct observation of one selected ICU per hospital. This form recorded data on cleanliness, organisation, care practices, frequency of patient monitoring and adherence to infection control measures.
- Patient and admission assessment form: collected patient-level data, including demographics and diagnoses, from the medical records of patients admitted on the day of the visit. For these patients, readmission was defined as any subsequent ICU admission during the same hospitalisation or after discharge, due to recurrence of the primary condition, comorbidities, complications from the prior ICU stay or new health issues, as identified through hospital records.

### Data collection preparation

A formal request was made to regional health administrators to recruit qualified data collectors with experience in ICU settings, such as emergency and critical care physicians, emergency and critical care nurses, anaesthesiologists and general practitioners. These individuals were assigned to conduct surveys in ICU facilities within their respective regions but were restricted from surveying hospitals where they were employed to avoid potential biases.

We used a 1-day snapshot approach across all facilities to ensure standardised, directly observed patient-level data collection at the national level. All patients admitted to the ICU on the observation day were included. In hospitals with more than one ICU, data collectors were instructed to observe the general ICU (or the largest mixed unit) for patient-level data, as this was most representative of case mix and routinely accessible. This pragmatic design reduced the reporting burden and improved data reliability by minimising reliance on incomplete or inconsistent retrospective records.

A training session was conducted for all data collectors and supervisors. During the training, participants were orientated to data collection procedures, familiarised with the survey variables and provided with a Standard Operating Procedure document. The session included discussions to clarify each variable and ensure consistency in data collection. To facilitate real-time communication and support, a dedicated Telegram group was created for all participants, easing communication and ensuring data quality throughout the process. The study coordinators further supervised data collection to ensure adherence to protocols, address challenges and provide ongoing support.

### Data quality control

Data entry and quality monitoring were conducted through a dedicated digital data collection platform, specifically designed for this study. Each regional and city administration health bureau focal person was granted access to oversee data entry for their respective regions. They ensured data completeness and accuracy by regularly reviewing submissions, while a national technical working group provided overarching supervision to ensure adherence to data collection protocols and maintain high-quality standards.

### Statistical analysis

Data were exported from the dedicated digital data collection platform to Excel and analysed using STATA V.17. Descriptive statistics were used to summarise facility characteristics, while comparative analyses were performed to contrast results from the pre-COVID-19 study with the current post-COVID-19 status; no multivariable analysis was performed. Comprehensive reports were generated for each hospital and each region, with results meticulously validated through cross-checks with regional health bureau representatives to ensure precision and reliability. Missing data were reported transparently and no imputation was performed.

### Supplementary data source

In addition to the survey dataset, we obtained national ICU performance indicators from the Ministry of Health’s District Health Information System V.2 (DHIS2) for the years 2021–2024. These included ICU deaths, the number of patients on mechanical ventilation, ICU discharges and ventilator-associated pneumonia (VAP) cases. The indicators were compiled from routine facility reporting and are presented in tabular form to provide a complementary national context alongside the survey findings. These variables were introduced into the DHIS platform beginning in 2021; before then, only ICU deaths and discharges were systematically recorded, so earlier data were excluded from this analysis. They were excluded from the primary analysis.

### Patient and public involvement

Patients and the public were not involved in the design, conduct, reporting or dissemination plans of this nationwide ICU capacity assessment. The study focused on institutional-level and system-level data collected through hospital visits and structured facility questionnaires, which did not directly involve individual patient experiences or require public input during development. However, the findings aim to inform future policy and advocacy efforts for equitable access to critical care, and dissemination strategies will prioritise sharing results with key stakeholder groups, including community health advocates and civil society organisations.

## Results

### General overview

**Figure 2:**
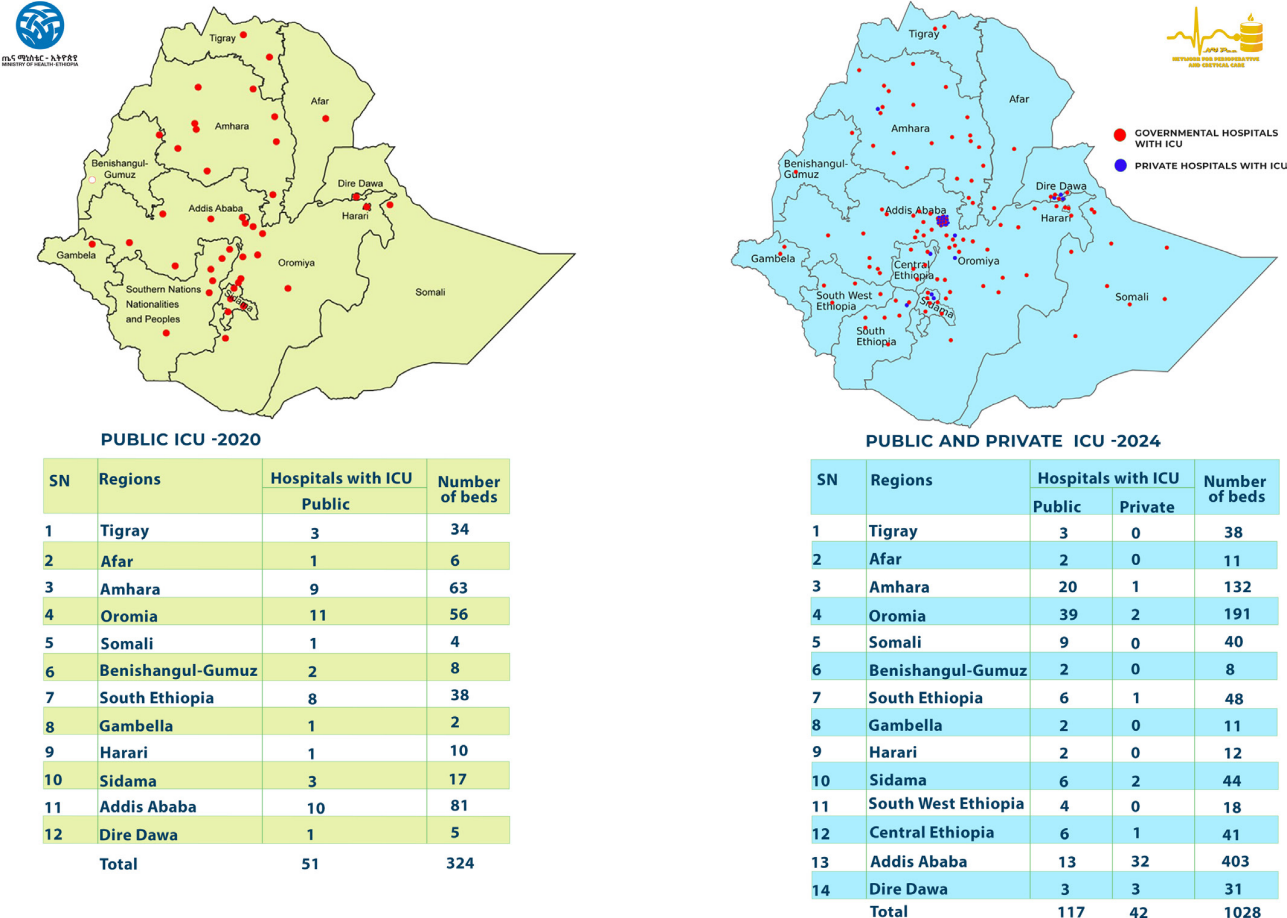
Distribution of intensive care units (ICUs) across regions before and after COVID-19*.*

As shown in figure 2, this study surveyed a total of 159 hospitals across all 14 regions and city administrations in Ethiopia, achieving a response rate of 93%. Twelve hospitals in Oromia Regional State were excluded due to instability in the region. Of the surveyed hospitals, 117/159 (73.58%) were governmental, while 42/159 (26.42%) were private facilities. A total of 190 ICUs were assessed, with a combined bed capacity of 1028 beds, ranging from two to 37 beds per ICU. The distribution of ICUs per facility varied: 136 had one ICU, 17 had two ICUs, four had three ICUs and two had four ICUs.

### Comparison of ICU and bed capacity pre-COVID-19 and post-COVID-19

As shown in figure 2 and online supplemental appendix B, there has been an increase in ICU facilities across Ethiopia since the onset of COVID-19, with hospitals with ICUs rising from 51 to 117 and total ICU beds increasing from 324 to 762, excluding private hospitals. Addis Ababa and Oromia saw the largest expansions, with ICU beds rising from 81 to 186 and 56 to 181, respectively. The Somali region showed significant improvement, with hospitals with ICUs increasing from one to nine and beds from four to 40, while Sidama doubled its hospitals with ICUs from three to six, with beds rising from 17 to 31. The regions previously grouped under Southern Nations, Nationalities and Peoples' Region of Ethiopia (SNNPR), now subdivided into three regions (Southwest, Central and South Ethiopia), collectively grew from eight hospitals with ICUs and 38 beds to 14 hospitals with ICUs and 116 beds. Conversely, regions like Benishangul-Gumuz showed no change, maintaining two hospitals with ICUs and eight beds.

10.1136/bmjgh-2025-021281.supp2 Supplementary data

### Comparison of service levels pre-COVID-19 and post-COVID-19

The comparison across the 12 domains highlights significant improvements in ICU capacity and services following the COVID-19 pandemic. Facilities with 24/7 ICU-trained physicians increased from 15/51 (29%) to 61/117 (52.13%), and advanced organ support resources, such as continuous renal replacement therapy (CRRT), were introduced in 9/117 (7.69%) of the surveyed ICUs (nine facilities). The number of hospitals offering ICU services increased from 51 to 117 (43.6%). Disaster preparedness plans increased from 3/51 (6%) to 25/117 (21.37%). Although some percentages declined, such as non-invasive monitoring (73% to 64.96%) and referral roles (61% to 32.48%), the actual number of facilities offering these services increased significantly. However, areas requiring further development include the availability of advanced organ support (limited to nine facilities), nurse-to-patient ratios (50 facilities not meeting a <1:2 ratio), regular educational engagement (nine facilities offering consistent programmes) and advanced haemodynamic monitoring, which remains available in only five facilities (4.27%), as further shown in tables 1 and 2.

**Table 1:** Comparison of service levels pre-COVID-19 and post-COVID-19 for each of the WFSICCM variables 1–6

Variable, level and criteria		No. of facilities, n (%)		No. of facilities increased
		Pre-COVID-19 (n=51)	Post-COVID-19 (n=117)	
**Availability of skilled medical personnel**				
1	Physicians with some critical care experience at least during the day. Variable access to other specialists	7 (14%)	36 (30.76%)	+29 facilities
2	Physicians with ICU training/experience available during the day and night. Ready access to other specialists	29 (57%)	20 (17.09%)	−9 facilities
3	Physicians with formal ICU training 24/7. Rapid access to a full complement of specialists	15 (29%)	61 (52.13%)	+46 facilities
**Nurse-to-patient ratio**				
1	Higher than the ward nurse-to-patient ratio	20 (39%)	16 (13.67%)	−4 facilities
2	Not <1:3	8 (16%)	51 (43.59%)	+43 facilities
3	Not <1:2	23 (45%)	50 (42.73%)	+27 facilities
**Availability of other specialist respiratory therapists, physiotherapists, nutritionists, etc**				
1	Other personnel available	22 (43%)	65 (55.55%)	+43 facilities
2	Variable inclusion of allied health personnel	21 (41%)	39 (33.33%)	+18 facilities
3	Allied health personnel as regular team members	8 (16%)	13 (11.11%)	+5 facilities
**Capacity to monitor acutely ill patients**				
1	Non-invasive or minimally invasive monitoring	37 (73%)	76 (64.96%)	+39 facilities
2	Invasive (blood pressure, central venous pressure), blood gas analysis	14 (28%)	36 (30.77%)	+22 facilities
3	Advanced haemodynamic monitoring (ultrasonography, cerebral, etc)	0 (0%)	5 (4.27%)	+5 facilities
**Availability of resources for the support of failing organ function**				
1	Capacity for oxygen therapy and non-invasive organ support	46 (90%)	54 (46.15%)	+8 facilities
2	Basic mechanical ventilatory and pharmacological cardiovascular support, intermittent RRT, nutrition	5 (10%)	54 (46.15%)	+49 facilities
3	Advanced ventilatory and haemodynamic support, continuous RRT, tracheostomy	0 (0%)	9 (7.69%)	+9 facilities
**Design and structure of the physical space**				
1	Dedicated geographical area	40 (78%)	115 (98.29%)	+75 facilities
2	Dedicated area with a central monitoring station	10 (20%)	0 (0%)	−10 facilities
3	Dedicated area, individual patient areas and a central monitoring station	1 (2%)	2 (1.7%)	+1 facility

No. of facilities increased, some have negative means, they have moved to the next level.

ICU, intensive care unit; RRT, renal replacement therapy; WFSICCM, World Federation of Societies of Intensive and Critical Care Medicine.

**Table 2:** Comparison of service levels pre-COVID-19 and post-COVID-19 for each of the WFSICCM variables 7–12

Variable, level and criteria		No. of facilities, n (%)		No. of facilities increased
		Pre-COVID-19 (n=510)	Post-COVID-19 (n=117)	
**Integration with ICU outreach services**				
1	Defined geographical area only	0 (0%)	17 (14.52%)	+17 facilities
2	Ad hoc interactions with other care areas	51 (100%)	50 (42.73%)	−1 facility
3	Outreach team, step-down, close collaboration with other care areas	0 (0%)	50 (42.73%)	+50 facilities
**Presence of formal educational and professional development services for staff**				
1	Variable engagement in continuing education	31 (60%)	65 (55.55%)	+34 facilities
2	Engagement in continuing education	13 (26%)	43 (36.75%)	+30 facilities
3	Regular engagement in continuing education	7 (14%)	9 (7.69%)	+ 2 facilities
**Presence of dedicated house staff and role as a centre for training expert personnel**				
1	Experienced nursing care 24/7. Ad hoc educational activity	43 (84%)	62 (52.99%)	+19 facilities
2	Nurses with extra training in critical care provide 24/7 care Organised educational activities for staff	7 (14%)	38 (32.47%)	+31 facilities
3	Nursing staff with specialist ICU training provide 24/7 care Formal educational programme for staff	1 (2%)	17 (14.53%)	+16 facilities
**Capacity for research and quality improvement activities**				
1	Basic quality improvement programme	39 (77%)	51 (43.59%)	+12 facilities
2	Formal quality improvement programme. Ad hoc research	8 (16%)	56 (47.86%)	+48 facilities
3	Formal education and quality improvement programme. Active research	4 (8%)	10 (8.55%)	+6 facilities
**Role in acting as a referral service for the hospital, the community and the country**				
1	Ad hoc. Policy for transfer to a higher ICU	0 (0%)	39 (33.33%)	+39 facilities
2	Resource for critically ill patients within the hospital	20 (39%)	40 (34.20%)	+20 facilities
3	Referral resource for other hospitals	31 (61%)	38 (32.48%)	+7 facilities
**Ability to scale up services in response to a disaster or a pandemic outbreak**				
1	Responsive in a disaster	32 (63%)	51 (43.59%)	+19 facilities
2	Resource for critically ill patients within the hospital	16 (31%)	41 (35.04%)	+25 facilities
3	Disaster preparedness plan and capacity	3 (6%)	25 (21.37%)	+22 facilities

No. of facilities increased, some have negative means, they have moved to the next level.

ICU, intensive care unit; WFSICCM, World Federation of Societies of Intensive and Critical Care.

### Current status of private facilities

Most private ICUs are concentrated in Addis Ababa (32/42; 76.2%), where 21 private hospitals offer ICU services, compared with 13 in the public sector. Across other regions, the presence of private ICUs is minimal. In six regions with no private ICUs, where public facilities provide the majority of ICU services, the overall private bed capacity is 266/1028 (25.88%) of the national bed capacity.

A high proportion of private facilities (37/42, 88.09%) have physicians with formal ICU training available 24/7, while a significant number (27/42, 64.29%) provide basic mechanical and pharmacological support for organ function. However, advanced organ support resources and haemodynamic monitoring are limited, with only 3/42 (7.14%) offering such capabilities. Many private facilities demonstrate strong dedication to education and quality improvement: 10/42 (23.81%) are participating in ongoing education, and 27/42 (64.28%) are implementing basic quality improvement programmes. Only 2/42 (4.76%) of facilities provide formal ICU training programmes for their staff. Integration with ICU outreach services is moderate, with 24/42 (57.14%) collaborating with other care areas. In 2/42 (4.76%) of facilities, disaster preparedness plans are in place and 5/42 (11.90%) of these facilities serve as referral resources for other hospitals, as shown in online supplemental appendix C.

10.1136/bmjgh-2025-021281.supp3 Supplementary data

### Patient-level information and ICU utilisation

Among the 279 ICU admissions assessed, 218/279 (78.1%) were from the public sector and 61/279 (21.9%) from the private sector. The patients were mainly from Addis Ababa (79/279, 28.3%), Oromia (64/279, 22.9%) and Amhara (45/279, 16.1%) regions. Additionally, 29 hospitals (18.2%) reported no admitted patients on the day of assessment. The mean age of patients was 39.1 years and males accounted for 154/279 (55.2%) of patients. The primary causes of ICU admission were neurological conditions (32.1%, 87/271), respiratory conditions (25.8%, 70/271) and cardiovascular conditions (10.3%, 28/271), with each patient assigned a single primary diagnosis to avoid overlap. Sepsis was observed as a secondary condition in 82/279 (29.4%) of all admitted patients, regardless of primary diagnosis. Hypertension (70/279, 25.1%) and diabetes mellitus (48/279, 17.2%) were the most frequent comorbidities, with higher rates of diabetes mellitus (17/48, 35.4%) and HIV (6/9, 66.7%) in patients with sepsis. Readmissions were low overall (15/279, 5.4%) among patients admitted to the ICU on the observation day and comparable among patients with sepsis (4/81, 4.9%), as shown in table 3.

**Table 3:** Patient-level information and ICU utilisation

Patients	All patients (n=279)	Patients with sepsis (n=82)	Patients without sepsis (n=197)
Age	39.1 (21.8)	40.4 (23.9)	38.6 (20.9)
Sex			
Male	154/279 (55.2%)	47/82 (57.3%)	107 (54.3%)
Female	125/279 (44.8%)	35/82 (42.7%)	90/197 (45.7%)
Readmission			
Yes	15/279 (5.4%)	4/81 (4.9%)	11/197 (5.6%)
No	263/279 (94.6%)	77/81 (95.1%)	186/197 (94.4%)
Primary indication for admission			
Neurological	87/271 (32.1%)	18/80 (22.5%)	69/191 (36.1%)
Respiratory	70/271 (25.8%)	21/80 (26.3%)	49/191 (25.7%)
Cardiovascular	28/271 (10.3%)	4/80 (5.0%)	24/191 (12.6%)
Trauma	28/271 (10.3%)	6/80 (7.5%)	22/191 (11.5%)
Gastrointestinal	14/271 (5.2%)	11/80 (13.8%)	3/191 (1.6%)
Genitourinary	13/271 (4.8%)	7/80 (8.8%)	6/191 (3.1%)
Metabolic/Endocrine	13/271 (4.8%)	2/80 (2.5%)	11/191 (5.8%)
Haematology	11/271 (4.1%)	8/80 (10.0%)	3/191 (1.6%)
Obstetrics	4/271 (1.5%)	2/80 (2.5%)	2/191 (1.1%)
Musculoskeletal	3/271 (1.1%)	1/80 (1.3%)	2/191 (1.1%)
Comorbidities			
Hypertension	70/279 (25.1%)	18/82 (22.0%)	52/197 (26.4%)
Diabetes mellitus	48/279 (17.20%)	17/82 (20.7%)	31/197 (15.7%)
Congestive heart failure	21/279 (7.5%)	4/82 (4.5%)	17/197 (8.6%)
COPD/Asthma	17/279 (6.1%)	6/82 (7.3%)	11/197 (5.6%)
Myocardial infarction	15/279 (5.4%)	4/82 (4.5%)	11/197 (5.6%)
Cerebrovascular accident/TIA	11/279 (3.9%)	5/82 (6.1%)	6/197 (3.1%)
Moderate-to-severe CKD	11/279 (3.9%)	7/82 (8.5%)	4/197 (2.0%)
HIV	9/279 (3.2%)	6/82 (7.3%)	3/197 (1.5%)
Connective tissue disease	8/279 (2.9%)	4/82 (4.5%)	4/197 (2.0%)
Liver disease	5/279 (1.8%)	2/82 (2.5%)	3/197 (1.5%)
Peripheral vascular disease	3/279 (1.1%)	2/82 (2.5%)	1/197 (0.5%)
Dementia	3/279 (1.1%)	3/82 (3.7%)	0/197 (0%)
Solid tumour	2/279 (0.7%)	2/82 (2.5%)	0/197 (0%)

CKD, chronic kidney disease; COPD, chronic obstructive pulmonary disease; ICU, intensive care unit; TIA, transient ischaemic attack.

### Supplementary national ICU performance indicators (2021–2024)

National DHIS data showed that ICU discharges and the number of patients on mechanical ventilation increased from 2021, peaking in 2023 (40 992 discharges; 11 730 ventilated patients), before declining in 2024. ICU deaths also rose during 2022–2023 but dropped substantially in 2024. Reported VAP cases varied, with the highest burden in 2022. These trends provide complementary insight into national ICU utilisation and outcomes (online supplemental appendix D).

10.1136/bmjgh-2025-021281.supp4 Supplementary data

## Discussion

This nationwide assessment of ICU capacity and utilisation across 159 Ethiopian hospitals shows substantial gains in critical care infrastructure and human resources. ICU bed capacity tripled from 324 to 1028 beds, and hospitals offering ICU services increased from 51 to 117, raising national ICU density from 0.3 to 0.9 beds per 100 000 population. Although still below the global average of 5–30 beds per 100 000, this rapid expansion represents a promising step in strengthening Ethiopia’s critical care system and offers a potential model for scaling ICU capacity in other low-resource settings. This progress builds on existing initiatives like HSDP and SaLTS, which laid the groundwork before COVID-19. The pandemic accelerated reforms, with about US$154 million allocated to health system strengthening, including ICU infrastructure, oxygen plants and workforce training.

The regional distribution of ICUs in Ethiopia has also improved after COVID-19, with more growth in Addis Ababa, Oromia, Somali, Sidama and the restructured southern regions, reflecting a move towards decentralising critical care compared with the previous assessment.^14^ This trend aligns with findings from big African regional studies, which emphasised that regional disparities in ICU access and outcomes are common across Africa and that expanding services beyond urban hubs is critical for improving survival in critically ill patients.^13 21 22^ However, regions such as Benishangul-Gumuz have seen no change, highlighting persistent inequities also noted in Nigerian and Ugandan assessments, where ICU resources remain concentrated in major cities.^22 23^ These disparities underscore the need for targeted regional investment and support for essential emergency and critical care (EECC) in all facilities, as advocated by EECC consensus recommendations and WHO-aligned system reviews.^6 24^

From a service readiness perspective, this study documented significant post-COVID-19 improvements. The availability of 24/7 ICU-trained physicians increased from 29% to 52.1%, and disaster preparedness plans rose from 6% to 21.4% of public facilities. This improvement likely stems from the MOH’s collaboration with WHO and other stakeholders, which helped to recruit 45 000 temporary staff and the training programmes being implemented to provide timely training programmes for over 2500 clinicians, significantly strengthening the availability of a trained healthcare workforce in the ICUs.^25–27^

Despite this progress, the study highlights ongoing limitations in advanced monitoring and organ support. Only 5% of facilities offer invasive haemodynamic monitoring or CRRT. These constraints are consistent with findings from both the African COVID-19 Critical Care Outcomes Study (ACCCOS) and the African Critical Illness Outcomes Study (ACIOS), which reported widespread deficits in access to advanced technologies, including vasopressors, mechanical ventilation and renal replacement therapy across African ICUs.^13 21 28^ Contributing factors include prohibitive costs, limited infection control infrastructure and a shortage of trained personnel, which continue to undermine complex critical care delivery in resource-constrained settings.^29–31^ While such resources are essential for managing circulatory collapse and improving outcomes in septic shock, accounting for 29% of ICU admissions in this study, they remain inaccessible to most facilities.^32 33^ Non-invasive technologies, such as continuous cardiac output monitoring and bedside ultrasound, have shown promise in other low-resource contexts as safer, scalable and cost-effective alternatives, warranting implementation research and policy attention in the Ethiopian setting.

A unique strength of this study is its inclusion of private healthcare facilities, which contribute 10.8% of the nation’s ICU bed capacity. Predominantly located in Addis Ababa, these facilities generally demonstrate stronger specialist coverage and greater engagement in quality improvement initiatives compared with public institutions. However, both sectors face persistent constraints in disaster preparedness, advanced monitoring and structured referral systems. These challenges are compounded by weak public-private integration, limited joint training opportunities and inconsistent adherence to national referral protocols, issues echoed in broader health systems reviews across sub-Saharan Africa that emphasise the critical role of formalised public-private partnerships in promoting equity, efficiency and service quality in critical care delivery.^6 29 31^

Beyond capacity metrics, this study reveals ICU patient characteristics: a young group with a mean age of 39.1 years, male predominance (55.2%) and common admission diagnoses, including neurological (32.1%), respiratory (25.8%) and cardiovascular (10.3%). The predominance of young, critically ill patients is consistent with the Ethio-SOS, which demonstrated high perioperative complication rates and notable ICU utilisation among surgical patients, showing the broader vulnerability of younger populations to critical illness in Ethiopia.^17^ These findings also align with reports from Uganda and Tanzania, as well as multicountry studies such as ACCCOS and ACIOS, which similarly highlight the high burden of sepsis and non-communicable complications among ICU patients in Africa.^13 21 23 34^ The increased readmission rate among patients with sepsis shows the systemic gaps in infection prevention, antimicrobial stewardship and post-ICU continuity of care. Importantly, the study reinforces the global call, advanced by the EECC initiative and the ACCCOS findings, that life-saving interventions such as oxygen therapy, intravenous fluids and airway management should be universally available, not restricted to high-tech ICUs.^10 13 21^

Trauma accounted for only a small proportion of ICU admissions in our study, despite evidence that Ethiopia bears a high injury burden, with an age-standardised incidence exceeding 7000 per 100 000 in 2019.^35^ This discrepancy likely reflects referral and admission practices, where many trauma patients are managed outside ICUs, often in surgical or emergency wards, particularly in facilities without neurosurgical or advanced trauma capacity.^36^ By contrast, the predominance of sepsis, neurological and respiratory conditions, alongside comorbidities such as hypertension and diabetes, is consistent with Ethiopia’s epidemiological transition, where non-communicable diseases increasingly intersect with acute critical illness.^37^ These findings highlight the need to scale up EECC to ensure equitable access to life-saving interventions, while continuing to expand ICU capacity for patients requiring advanced monitoring and organ support.^24^

In addition, routine DHIS data from 2021 to 2024 showed rising ICU discharges and mechanical ventilation cases, which peaked in 2023 and declined in 2024, with deaths following a similar surge-and-drop pattern. These patterns are consistent with the COVID-19 surge and stabilisation of critical-care demand, supported by a systematic review showing a 14.4% pooled COVID-19 mortality in Ethiopia, particularly high among patients with comorbidities,^38^ further underscoring the increased burden faced by ICUs during this period. However, as DHIS reporting primarily reflects public facilities, these findings should be interpreted cautiously, with private ICUs likely under-represented. This underscores the need for a comprehensive ICU surveillance system that includes private facilities and incorporates validated quality metrics, as recently advocated in LMIC settings,^39^ to strengthen comparability and guide policy.

The main limitation of this study is that it does not assess patient outcomes, as its primary aim was to describe admission demographics and ICU capacity. In addition, some facilities were excluded due to logistical challenges, which may introduce selection bias and slightly limit the comprehensiveness of the data. In hospitals with multiple ICUs, only one unit was observed for patient-level data, potentially underestimating the case mix across all units. The use of a 1-day snapshot, while ensuring feasibility and standardised direct observation across 159 hospitals, may not fully reflect seasonal or cultural variations in admission patterns. Finally, the supplementary DHIS data were reported directly by facilities without independent validation, raising the possibility of reporting bias and incomplete reporting. Despite these limitations, the study effectively addresses its primary research questions and provides a robust, comprehensive foundation for future research, offering insights to guide improvements in critical care services and inform evidence-based interventions.

In conclusion, this nationwide assessment demonstrates that Ethiopia’s critical care landscape has undergone marked expansion over the past 5 years, with ICU capacity nearly tripling, regional distribution improving and human resources becoming more readily available. The inclusion of private facilities and stronger national coordination reflects the system’s increasing maturity. Yet, persistent gaps in monitoring, advanced organ support and emergency readiness highlight the limits of this growth. To build a resilient and equitable critical care system, Ethiopia must now shift from rapid expansion to strategic consolidation, anchoring future investments in EECC, structured public-private integration, routine ICU surveillance systems to guide decision-making, and strengthened infection management, including antimicrobial stewardship, alongside context-specific implementation research. These lessons are relevant not only to Ethiopia but also to comparable settings across sub-Saharan Africa and other low-resource contexts facing similar challenges.

10.1136/bmjgh-2025-021281.supp1 Supplementary data

10.1136/bmjgh-2025-021281.supp5 Supplementary data

10.1136/bmjgh-2025-021281.supp6 Supplementary data

## Data Availability

Data are available on reasonable request. The data used in this study will be available on request. All shared data will be anonymised to ensure that no identifiable information is disclosed. For requests, please contact us at Info@n4pcc.com. There is no fixed end date for data availability.
