# Supplementary material for: Critical care capacity in Africa: postpandemic ICU capacity, service readiness and patient profiles across public and private hospitals in Ethiopia
Source: BMJ Glob Health. 2026 Mar 24;11(3):e021281. doi: 10.1136/bmjgh-2025-021281 (PMC13157738; doi:10.1136/bmjgh-2025-021281)
Supplement: Supplementary data [file bmjgh-11-3-s002.pdf]

Appendix B

Appendix B1: Current Regional Distribution of ICUs, and ICU Beds

Regions	Hospitals with ICU		Total number of ICUs	Total beds (range/ICU)	Number of patients in the survey
	Governmental	Private			
Addis Abeba	13	32	45	403(2-37)	79
Oromia	39	2	41	191(2-24)	64
Amhara	20	1	21	132(2-26)	45
Southwest Ethiopia	4	0	4	18(4-6)	11
Central Ethiopia	6	1	7	41(2-11)	10
South Ethiopia	6	1	7	48(5-12)	15
Tigray	3	0	3	38(6-24)	7
Sidama	6	2	8	44(4-10)	15
Benishangul-Gumuz	2	0	2	8(4)	3
Afar	2	0	2	11(4-7)	4
Dire Dawa	3	3	6	31(3-7)	10
Gambella	2	0	2	11(5-6)	4
Harari	2	0	2	12(4-8)	4
Somali	9	0	9	40(2-11)	8
**Total**	**117**	**42**	**159**	**1028(2-37)**	**279**

Abbreviations: ICU; intensive care unit.
