# Supplementary material for: Critical care capacity in Africa: postpandemic ICU capacity, service readiness and patient profiles across public and private hospitals in Ethiopia
Source: BMJ Glob Health. 2026 Mar 24;11(3):e021281. doi: 10.1136/bmjgh-2025-021281 (PMC13157738; doi:10.1136/bmjgh-2025-021281)
Supplement: Supplementary data [file bmjgh-11-3-s003.pdf]

Appendix: C

Table C1: Current Status of Private Facilities for Each of the WFSICCM Variables 1–6

Variable, level, and criteria		No. of Private facilities n (%) (n=42)
Availability of skilled medical personnel		
1	Physicians with some critical care experience at least during the day. Variable access to other specialists	3(7.14%)
2	Physicians with ICU training/experience available during day and night. Ready access to other specialists	2(4.76%)
3	Physicians with formal ICU training 24/7. Rapid access to full complement of specialists	37(88.09%)
Nurse to patient ratio		
1	Higher than ward nurse to patient ratio	1(2.38%)
2	Not <1:3	17(40.47%)
3	Not <1:2	24(57.14%)
Availability of other specialists—respiratory therapists, physiotherapists, nutritionists, etc.		
1	Other personnel available	10(23.81%)
2	Variable inclusion of allied health personnel	24(57.14%)
3	Allied health personnel as regular team members	8(19.04%)
Capacity to monitor acutely ill patients		
1	Noninvasive or minimally invasive monitoring	13(30.95%)
2	Invasive (blood pressure, central venous pressure), blood gas analysis	26(61.90%)
3	Advanced hemodynamic monitoring (ultrasonography, cerebral, etc)	3(7.14%)
Availability of resources for the support of failing organ function		
1	Capacity for oxygen therapy and noninvasive organ support	12(28.57%)
2	Basic mechanical ventilatory and pharmacological cardiovascular support, intermittent RRT, nutrition	27(64.29%)
3	Advanced ventilatory and hemodynamic support, continuous RRT, tracheostomy	3(7.14%)
Design and structure of the physical space		
1	Dedicated geographical area	30(71.42%)
2	Dedicated area with central monitoring station	3(7.14%)
3	Dedicated area, individual patient areas, and central monitoring station	9(21.42%)

Abbreviations: ICU: intensive care unit; RRT: renal replacement therapy; WFSICCM: World Federation of Societies of Intensive and Critical Care Medicine.

Table B2: Current of Private Facilities for Each of the WFSICCM Variables 7–12

Variable, level, and criteria		No. of Private facilities n (%)
Integration with ICU outreach services		
1	Defined geographical area only	4(9.52%)
2	Ad hoc interactions with other care areas	14(33.33%)
3	Outreach team, step-down, close collaboration with other care areas	24(57.14%)
Presence of formal educational and professional development services for staff		
1	Variable engagement in continuing education	31(73.81%)
2	Engagement in continuing education	10(23.81%)
3	Regular engagement in continuing education	1(2.38%)
Presence of dedicated house staff and role as a center for training expert personnel		
1	Experienced nursing care 24/7. Ad hoc educational activity	26(61.91%)
2	Nurses with extra training in critical care provide 24/7 care<br>Organized educational activities for staff	14(33.33%)
3	Nursing staff with specialist ICU training provide 24/7 care<br>Formal educational program for staff	2(4.76%)
Capacity for research and quality improvement activities		
1	Basic quality improvement program	27(64.28%)
2	Formal quality improvement program. Ad hoc research	11(26.19%)
3	Formal education and quality improvement program. Active research	4(9.52%)
Role in acting as a referral service for the hospital, the community, and the country		
1	Ad hoc. Policy for transfer to higher ICU	7(16.67%)
2	Resource for critical ill patients within hospital	30(71.43%)
3	Referral resource for other hospitals	5(11.90%)
Ability to scale-up services in response to disaster or pandemic outbreak		
1	Responsive in disaster	16(38.09%)

Table B2: Current of Private Facilities for Each of the WFSICCM Variables 7–12

Variable, level, and criteria		No. of Private facilities n (%)
2	Resource for critical ill patients within hospital	24(57.14%)
3	Disaster preparedness plan and capacity	2(4.76%)

Abbreviations: ICU: intensive care unit; WFSICCM: World Federation of Societies of Intensive and Critical Care
