# Supplementary material for: Critical care capacity in Africa: postpandemic ICU capacity, service readiness and patient profiles across public and private hospitals in Ethiopia
Source: BMJ Glob Health. 2026 Mar 24;11(3):e021281. doi: 10.1136/bmjgh-2025-021281 (PMC13157738; doi:10.1136/bmjgh-2025-021281)
Supplement: Supplementary data [file bmjgh-11-3-s004.pdf]

Appendix D

Table D1: National ICU Performance Indicators, 2021–2024

Year (GC)	ICU Deaths	Patients on Mechanical Ventilation	ICU Discharges	VAP Cases
2021	1329	912	29,075	122
2022	8776	7526	32,397	705
2023	10,372	11,730	40,992	568
2024	4861	5615	20,443	386

Note: Data sourced from the Federal Ministry of Health, Ethiopia.
