# Supplementary material for: Critical care capacity in Africa: postpandemic ICU capacity, service readiness and patient profiles across public and private hospitals in Ethiopia
Source: BMJ Glob Health. 2026 Mar 24;11(3):e021281. doi: 10.1136/bmjgh-2025-021281 (PMC13157738; doi:10.1136/bmjgh-2025-021281)
Supplement: Supplementary data [file bmjgh-11-3-s001.pdf]

Appendix A

Writing committee

Tesfay Yohannes MSc[1], Fitsum K Belachew PhD[2], Degisew Derso MSc[3], Selam Daniel MD[4], Azeb Demelash MPH[3], Kalkidan Kifle BSc[1], Wagari Tuli Nora MSc[6] Elubabor Buno Teko MD[3], Emnet Tesfaye MD[7], Yared Boru[2], Rupert Pearse PhD[8], and Menbeu Sultan MD[9]

on behalf of ENCCA collaborators

Affiliations:

1. Department of Statistical and Data Governance, Global Partners for Improving Surgical System (GPISS), Project Network for Perioperative and Critical Care (GPISS-N4PCc), Addis Ababa, Ethiopia.
2. Project Network for Perioperative and Critical Care (N4PCc), Debre Berhan University, Asrat Woldeyes Health Science Campus, Debre Berhan, Amhara region, Ethiopia
3. Medical Services, Ministry of Health, Addis Ababa, Ethiopia
4. Department of Anesthesia, St. Peter specialized hospital, Addis Ababa, Ethiopia.
5. Department of Emergency and Critical Care, Addis Ababa University Addis Ababa, Ethiopia.
6. Medical Services, Ministry of Health, Addis Ababa, Ethiopia
7. Emergency and Critical Care Medicine, Hawassa University, Hawassa, Ethiopia.
8. William Harvey Research Institute, Queen Mary University of London, London, UK
9. Department of intensive care, African health science university, Kigali, Rwanda

ENCCA collaborators

Addis Ababa City: Desta Kassaye (Regional coordinator), Kerebih Hailemariam, Workineh Aniley, Ali Seid, Chanyalew Abdeta, Moti Dejene, Yonas Belachew, Solomon Addise, Dereje Hailu, Yonatan Tesema, Kejela Mosisa, Eyuel Teshome, Ambawu Siyum, Gashaw W/kidan, H/mariam Zekarias, Melke Mare, Hirut Tolasa, Feregzh Dinku, and Abeba Tegegn.

Afar Region: Ali Mohammed (Regional coordinator), kedir Hussen, Ibrahim Aliye, Ahmed Adem.

Amhara Region: Andargie Atnaf (Regional coordinator), Sisay Nigusu, Kibret Engocha, Shumet Gathaye, Astazbew Tehuala, Siquar Zinabu, Getie Demelash, Getasew Misganaw, Dinku Yegnasew, Bruk Tsegaye, Wondissew Admasu, Habtamu Nahusenay, Begosew Yeshiwas, Awoke Warkaw, Asnakew Alem, Tesfaye Ayenew, Tirusew Getie, and Kemal Adem.

Benishangul Gumuz Region: Biruktie GebreAregawi (Regional coordinator), Biftu Gudisa, and Tariku Lenchamo.

Central Ethiopia: Tadele Temesgen (Regional coordinator)

Diredawa Region: Yared Hailu (Regional coordinator), Zelalem Nigussie, Eyuel Tewodros.

Gambella Region: Teka Kemal (Regional coordinator), Ashanif Temsgne, and Solomon Abera.

Harar Region: Yonas Alamrew (Regional coordinator), Biruk Tizazu, Seid Ali, and Beyazilign Alemu.

Oromia Region: Kananisa Layo (Regional coordinator), Bayisa Guteta, Amde Getahun, Girma Mesfin, Tamirat Tesfaye, Garoma Gamachu, Feleke Ledago, Gamachis Amenu, Dawit Legesse, and Canyalew Abdeta.

Somali Region: Abdikadir A. Abdi (Regional coordinator), Nimcan A. Abdi, Salamoon warqu, Hassen A. Hussein,

Sidama Region: Asaminew Tasew (Regional coordinator), kindalem Worku, Aytnew Debebe, Tariku Tadese, Getachew Samuel

South Ethiopia: Woineshet Akinaw (Regional coordinator), Wondimu Dori, Ashenafi Anjulo, and Brihanu Anjulo.

South-west Region: Edgete Lemma (Regional coordinator), Alemayew Yesehaq, Doctor Antenhe Berhane.

Tigray Region: Tewodros Gobezay (Regional coordinator), Tsegay G/Mikael, Merhawit Abreha, Yilikal Gebru
