# Supplementary material for: Critical care capacity in Africa: postpandemic ICU capacity, service readiness and patient profiles across public and private hospitals in Ethiopia
Source: BMJ Glob Health. 2026 Mar 24;11(3):e021281. doi: 10.1136/bmjgh-2025-021281 (PMC13157738; doi:10.1136/bmjgh-2025-021281)
Supplement: Supplementary data [file bmjgh-11-3-s005.pdf]

Appendix E- STROBE checklist

STROBE Statement- Checklist of items that should be included in reports of ***cross-sectional studies***

	Item No	Recommendation	Yes/No
**Title and abstract**	1	(*a*) Indicate the study's design with a commonly used term in the title or the abstract	Title page
		(*b*) Provide in the abstract an informative and balanced summary of what was done and what was found	Abstract
**Introduction**			
Background/rationale	2	Explain the scientific background and rationale for the investigation being reported	Background, pp. 3–4
Objectives	3	State specific objectives, including any prespecified hypotheses	End of Background, p. 4
**Methods**			
Study design	4	Present key elements of study design early in the paper	Methods > Study design, p. 6
Setting	5	Describe the setting, locations, and relevant dates, including periods of recruitment, exposure, follow-up, and data collection	Methods > Setting & data collection, pp. 6–7
Participants	6	(*a*) Give the eligibility criteria, and the sources and methods of selection of participants	Methods > Participants, p. 6-7
Variables	7	Clearly define all outcomes, exposures, predictors, potential confounders, and effect modifiers. Give diagnostic criteria, if applicable	Methods > Study tool design & Patient/admission assessment, pp. 6–8
Data sources/ measurement	8*	For each variable of interest, give sources of data and details of methods of assessment (measurement). Describe comparability of assessment methods if there is more than one group	Methods > Data sources & DHIS, pp. 6–7
Bias	9	Describe any efforts to address potential sources of bias	Methods > Data collection prep & Limitations, pp. 6, 20 (training, SOPs, non-self facilities, DHIS reporting bias, snapshot bias)
Study size	10	Explain how the study size was arrived at	Methods > Census approach, p. 7 (all ICUs with minimum WFICC definition included; no sample size calc)
Quantitative variables	11	Explain how quantitative variables were handled in the analyses. If applicable, describe which groupings were chosen and why	Methods > Statistical analysis, p. 7

Belachew FK, *et al. BMJ Glob Health* 2026; 11:e021281. doi: 10.1136/bmjgh-2025-021281

Statistical methods	12	(*a*) Describe all statistical methods, including those used to control for confounding	Methods > Statistical analysis, p. 7
		(*b*) Describe any methods used to examine subgroups and interactions	p. 7
		(*c*) Explain how missing data were addressed	N/A
		(*d*) If applicable, describe analytical methods taking account of sampling strategy	N/A
		(*e*) Describe any sensitivity analyses	N/A
**Results**			
Participants	13*	(a) Report numbers of individuals at each stage of study, e.g., numbers potentially eligible, examined for eligibility, confirmed eligible, included in the study, completing follow-up, and analysed	Results > General overview, pp. 8–9
		(b) Give reasons for non-participation at each stage	N/A
		(c) Consider use of a flow diagram	Result/ Figure 1
Descriptive data	14*	(a) Give characteristics of study participants (eg demographic, clinical, social) and information on exposures and potential confounders	Results > Patient-level info, pp. 12–18, Table 3
		(b) Indicate number of participants with missing data for each variable of interest	Results/Table 1
Outcome data	15*	Report numbers of outcome events or summary measures	Results > Patient-level info, pp. 12–18
Main results	16	(*a*) Give unadjusted estimates and, if applicable, confounder-adjusted estimates and their precision (eg, 95% confidence interval). Make clear which confounders were adjusted for and why they were included	Results > Tables 1–3, pp. 12–18
		(*b*) Report category boundaries when continuous variables were categorized	N/A
		(*c*) If relevant, consider translating estimates of relative risk into absolute risk for a meaningful time period	N/A
Other analyses	17	Report other analyses done—eg analyses of subgroups and interactions, and sensitivity analyses	N/A
**Discussion**			
Key results	18	Summarise key results with reference to study objectives	Discussion, p. 19
Limitations	19	Discuss limitations of the study, taking into account sources of potential bias or imprecision. Discuss both direction and magnitude of any potential bias	Discussion > Limitations, pp. 21–22
Interpretation	20	Give a cautious overall interpretation of results considering objectives, limitations, multiplicity of analyses, results from similar studies, and other relevant evidence	Discussion, pp. 16–20

Belachew FK, *et al. BMJ Glob Health* 2026; 11:e021281. doi: 10.1136/bmjgh-2025-021281

Generalisability	21	Discuss the generalisability (external validity) of the study results	Discussion > final para, p. 21 (national representativeness; relevance to other low-resource settings)
**Other information**			
Funding	22	Give the source of funding and the role of the funders for the present study and, if applicable, for the original study on which the present article is based	Funding statement, p. 23
Ethics		Ethical approval, consent	Methods > Ethical approval, p. 9

*Give information separately for exposed and unexposed groups.

Note: An Explanation and Elaboration article discusses each checklist item and gives methodological background and published examples of transparent reporting. The STROBE checklist is best used in conjunction with this article (freely available on the websites of PLoS Medicine at http://www.plosmedicine.org/, Annals of Internal Medicine at http://www.annals.org/, and Epidemiology at http://www.epidem.com/). Information on the STROBE Initiative is available at www.strobe-statement.org.

Belachew FK, *et al. BMJ Glob Health* 2026; 11:e021281. doi: 10.1136/bmjgh-2025-021281
