# Supplementary material for: Critical care capacity in Africa: postpandemic ICU capacity, service readiness and patient profiles across public and private hospitals in Ethiopia
Source: BMJ Glob Health. 2026 Mar 24;11(3):e021281. doi: 10.1136/bmjgh-2025-021281 (PMC13157738; doi:10.1136/bmjgh-2025-021281)
Supplement: Supplementary data [file bmjgh-11-3-s006.pdf]

BMJ Global Health Author Reflexivity Statement

Adapted from Morton, B., Vercueil, A., Masekela, R., Heinz, E., Reimer, L., Saleh, S., Kalinga, C., Seekles, M., Biccard, B., Chakaya, J., Abimbola, S., Obasi, A. and Oriyo, N. (2022), Consensus statement on measures to promote equitable authorship in the publication of research from international partnerships. Anaesthesia, 77: 264-276. https://doi.org/10.1111/anae.15597

**Study conceptualisation**	
1. How does this study address local research and policy priorities?	This study addresses nationally identified priorities related to strengthening emergency and critical care services in Ethiopia, particularly following COVID-19. It aligns with Ministry of Health strategies on intensive care expansion, oxygen systems, workforce development, and Essential Emergency and Critical Care (EECC), and responds to gaps highlighted by prior national assessments that lacked patient-level and private-sector data.
2. How were local researchers involved in study design?	The study was conceptualised and designed by Ethiopia-based researchers in collaboration with the Ministry of Health. Local clinicians, health system leaders, and international academic partners shaped the study questions, tools, and design to ensure relevance to national service delivery realities and feasibility across diverse regional contexts.
**Research management**	
3. How has funding been used to support the local research team(s)?	Funding supported national coordination, development of data collection tools, training of Ethiopian data collectors, and implementation of a secure digital data platform. Resources were directed toward strengthening local research capacity rather than external data collection or analysis.
**Data acquisition and analysis**	
4. How are research staff who conducted data collection acknowledged?	Data collection was conducted by Ethiopian clinicians (including emergency and critical care physicians, anaesthetists, and nurses) recruited through regional health bureau. Their contributions are acknowledged through formal collaborator recognition and institutional reporting.
5. How have members of the research partnership been provided with access to study data?	Data access was governed through Ministry of Health oversight and ethical approval. Ethiopia-based investigators had full access to cleaned and linked datasets for analysis and interpretation, with data sharing aligned to national data governance and confidentiality standards.
6. How were data used to develop analytical skills within the partnership?	Local researchers led data cleaning, linkage, descriptive analysis, and interpretation, supported by collaborative methodological discussions in consultation with an international academic partner. The study contributed to strengthening

	skills in health systems analytics, the use of registry-linked data, and cross-facility quality assessment.
**Data interpretation**	
7. How have research partners collaborated in interpreting study data?	Findings were interpreted through joint discussions involving ministry representatives, local clinicians, and academic partners. Interpretation emphasised policy relevance, feasibility, and system-level implications for Ethiopia and similar African settings.
**Drafting and revising for intellectual content**	
8. How were research partners supported to develop writing skills?	Ethiopia-based authors led manuscript drafting and revision. Iterative internal reviews and collaborative editing supported capacity development in scientific writing, peer-review response, and international journal submission.
9. How will research products be shared to address local needs?	Findings will be shared with the Ministry of Health, regional health bureau, and participating facilities through reports, presentations, and policy discussions to inform planning for ICU expansion, EECC scale-up, and referral system strengthening.
**Authorship**	
10. How is the leadership, contribution and ownership of this work by LMIC researchers recognised within the authorship?	The study is led by Ethiopia-based investigators, with first and senior authorship held by local researchers. Authorship reflects substantive contributions to study design, data collection, analysis, interpretation, and manuscript preparation.
11. How have early career researchers across the partnership been included within the authorship team?	Early-career Ethiopian clinicians and researchers participated in data collection, analysis, and manuscript development and are included as authors or collaborators in line with contribution.v
12. How has gender balance been addressed within the authorship?	The authorship team includes women and men across clinical, academic, and policy roles, reflecting efforts to promote inclusive participation within national health research.
**Training**	
13. How has the project contributed to training of LMIC researchers?	The project provided structured training in data collection, use of digital platforms, standardised ICU assessment, and collaborative analysis, contributing to sustainable national research capacity in critical care.
**Infrastructure**	
14. How has the project contributed to improvements in local infrastructure?	The study strengthened national data infrastructure through a dedicated ICU survey platform, improved linkage with DHIS2 indicators, and enhanced systems for ongoing ICU monitoring and quality improvement.

Governance	
15. What safeguarding procedures were used to protect local study participants and researchers?	The study received ethical approval from an Ethiopian Institutional Review Board. Data were collected without patient identifiers, informed consent was waived due to the observational design, and researchers were protected through institutional oversight, ethical training, and clear data governance arrangements.
